# Highway to (Digital) Surveillance: When Are Clients Coerced to Share Their Data with Insurers?

**DOI:** 10.1007/s10551-020-04668-1

**Published:** 2020-11-08

**Authors:** Michele Loi, Christian Hauser, Markus Christen

**Affiliations:** 1 Institute of Biomedical Ethics and the History of Medicine, University of Zurich, Zurich, Switzerland; 2 PRME Business Integrity Action Center, University of Applied Sciences of the Grisons, Chur, Switzerland; 3 Digital Society Initiative, University of Zurich, Zurich, Switzerland

**Keywords:** Insurance, Threats, Coercion, Big data, Data sharing

## Abstract

Clients may feel trapped into sharing their private digital data with insurance companies to get a desired insurance product or premium. However, private insurance must collect some data to offer products and premiums appropriate to the client’s level of risk. This situation creates tension between the value of privacy and common insurance business practice. We argue for three main claims: first, coercion to share private data with insurers is *pro tanto* wrong because it violates the autonomous choice of a privacy-valuing client. Second, we maintain that irrespective of being coerced, the choice of accepting digital surveillance by insurers makes it harder for the client to protect his or her autonomy (and to act spontaneously and authentically). The violation of autonomy also makes coercing customers into digital surveillance *pro tanto* morally wrong. Third, having identified an economically plausible process involving no direct coercion by insurers, leading to the adoption of digital surveillance, we argue that such an outcome generates further threats against autonomy. This threat provides individuals with a *pro tanto* reason to prevent this process. We highlight the freedom dilemma faced by regulators who aim to prevent this outcome by constraining market freedoms and argue for the need for further moral and empirical research on this question.

## Introduction

Insurance is a genuinely data-driven industry and shows a keen interest in many applications of big data analytics and artificial intelligence, such as telematics in car insurance, fraud detection capabilities, or quantified-self applications for health and life insurances. Those technical developments are triggering a transformation of the insurance industry and allow dealing with risks that were previously considered uninsurable, such as earnings losses and cash flow volatility. The availability of large amounts of data that can be used to assess, select, price, predict, and prevent risks is key in this development.

A concern in the public debate is the fear of losing privacy when more and more customer data are made available for risk modeling (and many more applications of big data-driven modeling). This article contributes to this debate by suggesting a model for an ethical assessment of privacy risks when insurance uses big data analytics from digital surveillance sources. The focus will be on private insurance for natural persons, not (compulsory) social insurance or insurance for companies. The insurance case is especially interesting because, unlike other commercial applications of big data analytics such as personalized marketing or pricing, insurance companies have an ethically justified need to obtain client data to calculate risk-adequate premiums and prevent moral hazard. This need makes it difficult to set the limits of privacy protection: obviously, one cannot simply demand that insurers stop analyzing *all* the data from their clients relevant for predicting the insured risk.^1^ So, where should one place the limit?

In this essay, we argue for three claims: first, coercion to share private digital data with insurers is *pro tanto* wrong. *Coercion,* which leads to the violation of the autonomy of the client, is wrong because it disrespects autonomy. Because coercion fails to respect the autonomy of the client, we maintain that this is *pro tanto* wrong even if the client (who accepted sharing data to avoid paying higher prices) eventually ends up with a life that is *better* in some respect (for example, healthier), thanks to the coerced choice.

Second, we maintain that sharing data for insurance surveillance in some domain (for example, social media) may reduce the client’s autonomy in that domain, may oppose the client’s domain-specific preferences for privacy, and may facilitate further coercion of the client.

Third, we argue that it is economically possible for a market to evolve in a direction deprived of privacy-friendly options, even in the absence of threats. This evolution exposes the client to coercion, generating further risk to the client, to be *pro tanto* wronged.

We conclude that within an ethical framework that values autonomy, a countervailing reason is needed to justify allowing insurers to coerce clients into accepting some form of surveillance from digital devices. Finally, even in the absence of direct coercion from insurers, governments intending to protect the conditions of autonomy have *pro tanto* reasons to regulate this market.

We develop our argument by first explaining our framing assumptions, scope, and methodology (“Motivation, Framing Assumptions, Scope, and Methodology” section). In “Threats, conditional warnings, and conditional offers” section, we discuss the distinction, fundamental for our argument, between what we call ‘threats,’ ‘warnings’ and ‘offers,’ based on Robert Nozick’s account of coercion (Nozick 1969). In “Distinguishing threats from warnings and offers in the insurance domain” section, we use these distinctions in describing different types of interactions between insurers and their clients. “Privacy, autonomy, and the harm of being coerced into surveillance” section explains different ways in which privacy relates to autonomy, highlighting the different ways in which threats, offers and warnings affect autonomy, spontaneity, and authenticity. “Privacy erosion from non-coercive insurance market transactions” section argues that even non-coercive market transactions between insurers and their clients, leading to the acceptance of digital surveillance, can be inimical autonomy, which provides a *pro tanto* reason to avoid them. “Further research” section highlights questions for further research opened by our analysis. There is a brief conclusion.

Our account analyses potential violations of autonomy and losses of clients’ freedom deriving from insurers’ psychological threats against clients who do not share their data or who do not want to adopt risk mitigation measures inferred from surveillance data*.* This autonomy-based argument vis-à-vis coercion is a significant innovation because it expands the set of *deontological* moral elements, such as transparency or informed consent, considered in the literature about privacy and big data (Alder 1998; Ashworth and Free 2006; Hoven et al. 2012; ICDPPC 2018; Privacy International & Article 19 2018; Christen et al. 2019; Jobin et al. 2019; Nill et al. 2019). No account of *coercion by psychological threats* is found in most ethical analyses of either privacy *or* personalized pricing (Seele et al. 2019). The analysis of the concept of psychological threats (or ‘threats’ in the following explanations; see also Sect. 2 for the definition of the term ‘threat’) is important because it provides a treatment of coercion, one of the most puzzling and controversial concepts in our moral repertoire. Conceptual puzzles about coercion are also widespread outside philosophy, for example, in business (Eabrasu 2019), law (Honoré 1990) and, particularly, in data protection law.^2^ We focus here on coercion involving “a form of interference that necessarily involves the use of conditional proposals that render certain options ineligible for rational choice” (Pugh 2020, p. 92). There are two reasons to focus on this form of coercion. First, it is philosophically interesting because in coercion by threats, the will of the agent whose autonomy is violated is actively, not passively, involved in generating the undesired outcome, and yet the action counts as non-autonomous. Second, coercion by threats is not as visible, easy to detect, and when it is not recognized as such, controversial, as coercion by direct physical force.

We stress, however, that our analysis of threats is not based on the claim that this is the only *right* approach, and we explore the potential for moral wrongness that even non-coercive market transactions involving digital surveillance generate. The paper concludes that non-coercive privacy erosion through accepted conditional offers and the subsequent market adaptations (that in our analysis produce conditional warnings) are also morally problematic when privacy losses expose the client to further coercion risks.

## Motivation, Framing Assumptions, Scope, and Methodology

The insurance industry operates in a highly regulated environment, constrained by privacy and data protection laws, such as the General Data Protection Regulation in the European Union, anti-discrimination law, and possible regulation of insurance premiums; regulations also differ largely across countries. However, ethical questions have emerged that go beyond the realm of legal compliance and can damage the reputation and ethical characteristics of insurance companies if left unattended. For example, is the datafication of insurance an offense on people’s privacy?

Traditional insurance data is about standard demographics and the kind of events where insurance must pay, for example, the number of previous car accidents with civil liability in car insurance or the nature of illnesses in health insurance. Insurers have used this type of information for more than a century to offer competitive prices to low-risk clients and avert adverse selection (Stiglitz 1983; Wilkie 1997; Heath 2007; Palmer 2007). Insurance companies increasingly use information from what we define as digital *surveillance*, namely information about behavioral features that may be predictive of the risk insured against, where the relevant information is presumptively contained by digital data, or more precisely, the client’s digital phenotype (Loi 2019)*.* A feature of digital surveillance-based methods is that they attempt to identify patterns relevant to risk in the digital phenotype; that is, all possible data associated with an individual provide potential proxies of his or her risk while lacking *direct* access to individual behavior, except as reflected in data. As Loi (2019) argues, when machine learning models are applied to data produced for other purposes, risk factors identification is heavily influenced by the availability of the data. Such availability is, in turn, heavily influenced by capabilities of data production (for example, what data already exist and can be easily collected) that are not initially designed for the sake of measuring insurance risk. Data from social media or fitness sensors might be used. Thus, what determines the types of behaviors and choices that get penalized or incentivized by such models is the nature of the data-generating infrastructure, at least as much as the causal mechanism linking behavior and risk.

For example, some reports suggest that the US-based life insurance company John Hancock intends to sell policies only to customers who use wearable devices and smartphones to track fitness and health (Barlyn 2018). Such information may substitute for or, more likely, complement traditional risk-relevant information such as age or gender. Although the distinction between a surveillance-based insurance product and a traditional product is not sharp, it will guide our reflection about the different ethical profiles of such products.

The current *insurtech* landscape, mainly driven by technology start-ups, is increasingly creating business model innovations that include *surveillance features*: vehicle telematics, environmental sensors, home security, wearables, chronic condition management, and preventative healthcare. In such policies, risk mitigation options may be considered for adjusting underwriting dynamically and personalizing premiums (Braun and Schreiber 2017). Consider, for example, the “pay-as-you-drive” business model for car insurance, whereby telematics devices in cars record drivers’ data from which temporal, geographic, distance, acceleration, and braking patterns can be inferred (Braun and Schreiber 2017). The analysis of these individual patterns helps the insurer to assess the client’s risk and charge an appropriate premium.

As anticipated, the analysis revolves around the morally charged concepts of threats and coercion. These are some of the most complex and controversial concepts in ethics, and the risk of equivocation is high. For rigor’s sake, we rely on a well-defined and widely discussed substantive philosophical account of coercion. We make the relationship between privacy, autonomy, well-being, and real freedom explicit.

The concept of coercion by threats is one of the hardest to analyze in terms of the involvement of the will of coerced. Few philosophers today discuss coercion understood as interventions in which the agent is clearly *acted upon* (as opposed to *forced to act*) (Pugh 2020, p. 91). It would be misleading to consider “coercion by threats” as analogous to *coercion by direct physical force*. Coercion by direct physical force is the kind of coercion that follows from directly applying physical force on individuals, as a police officer does when handcuffing a suspect. The idea of coercion by direct physical force is obviously irrelevant here because insurers do not apply direct physical force on their clients. Threatening physical harm may loosely be defined as coercion *by force*, but it is achieved by altering options: physical force is *threatened*, not *directly exercised* as in direct physical coercion. Consider, for example, a burglar who coerces someone to turn over a wallet by threatening to take the individual’s life. A victim who obeys has been coerced *by the threat,* however, not by direct physical force. The burglar need not even touch the victim; hence the coercion works psychologically, not physically. (We refer to threats that alter the subject’s options as psychological, in this sense.) In coercion by threats, the agent is always *forced to act*, not *acted upon.*^3^ The will of the coerced individual is *active,* not *passive,* and it eventually determines the action. This outcome is more puzzling from the point of view of voluntariness than a passive condition in which the will of the individual is not exercised at all.

Deception and manipulation, while arguably equally hard to define, are less puzzling from the point of view of autonomy. Deception and manipulation quite intuitively undermine the rationality of the agent’s decision making: deception, in so far as the agent is made to act upon false beliefs, and manipulation, in so far as the agent is made to act upon preferences that are not authentically the agent’s own (Faden and Beauchamp 1986; Pugh 2020).

In contrast, in coercion by threats, the coerced agent may also be well-informed and acting upon authentic personal preferences. For example, a subject who is told that he or she will be killed unless he or she hands over a wallet may be acting upon an authentic valuing of life above money.

Philosophical accounts need to wrestle with this paradoxical idea: how can an action be voluntary, aligned with the agent’s preferences, and yet not autonomous. A further constraint is the need to identify an Archimedean point between two logically possible extremes: (1) the view that regards the sharing of personal information as *coerced* whenever the data controller requests the data as *a condition* of providing a benefit of any kind to the data subject; (2) the arguably more common-sense view that regards the choice to share data as *uncoerced* whenever the subject can afford to act otherwise (for example, by avoiding the transaction with the data controller) without suffering *significant harm* as a result. Notice that what counts as “significant” is notoriously problematic (Wertheimer 2014). So, we need an account that avoids presupposing we agree on what it means to suffer “significant harm.”

Both Wertheimer’s and Nozick’s views (along with several variations and tweaks of such views in the literature) satisfy this constraint. Here we focus on one aspect of Robert Nozick’s philosophical account of coercion by threats (Nozick 1969). Nozick talks about a normally expected baseline and mentions two possible interpretations of *normally expected*. We shall call the first the *status quo* interpretation. It refers to what is statistically normal or what a reasonable individual would expect to happen by deeming it probable. In an unjust world, something can be normal in this sense and yet be immoral. The alternative is the normative interpretation that Nozick also explores (Nozick claims that both can be used, depending on the context)^4^ and that Wertheimer (2014) defends as being the *only* correct option. In the latter formulation, the baseline of coercion is defined by the moral rights of the target of the threat. Applied to the case at hand, the proposal that the client pay a higher premium to avoid data surveillance counts as a threat only if the insurer makes the recipient worse off than the recipient *ought to be,* given his or her moral rights (Wertheimer 2014).

Here, we follow Nozick while also arguing that a *status quo* baseline account is more suitable to this context. We find the normative baseline account to be problematic for an analysis of threats by private insurers. It seems safe for us to assume that most insurance clients do not have a moral right to obtain the products offered by private insurance at a given price (excluding health insurance covering important health needs from the scope of this argument).^5^ Adopting the Wertheimer’s view of threats makes it almost trivial to show that insurers who demand higher prices from clients not sharing data are not threatening the clients, and that the clients do not count as coerced when they share their data. The only realistic situation in which a client could accuse an insurer of having obtained data via a threat is when the insurer makes a promise to the client that the prices will be stable, which the insurer then violates. (Such a promise will create at least a *prima facie* moral entitlement to its fulfillment by the insurer.) That point of view clearly limits the range of coercive threats by insurers to very special cases and, in our view, makes an analysis of the conditionality of premiums with respect to data in insurance quite uninteresting.

We do not aim to bring the contemporary debate on the baseline for coercive threats to an end (Anderson 2017; Eabrasu 2019; Pugh 2020; Sachs 2013), but we observe that Wertheimer’s account is vulnerable to significant objections, and Nozick’s view is not. For example, Wertheimer’s account cannot explain the common-sense view that if a justly imprisoned prisoner is told that he or she will have entertainment privileges withdrawn if he or she tries to escape, this counts as a (potentially coercive) threat, even if no prisoner has a *right* to such privileges (Olsaretti 1998). Given the contextual justification provided above, exploring the Nozick approach appears justified.

## Threats, Conditional Warnings, and Conditional Offers

According to Nozick (1969, pp. 441–45),^6^ P coerces Q *if and only if*

1. *P* aims to keep *Q* from choosing to perform action *A*;
2. *P* communicates a claim to *Q*;
3. *P*’s claim indicates that if *Q* performs *A*, then *P* will bring about some consequence that would make *Q*’s *A*-ing less desirable to *Q* than *Q*’s not *A*-ing;
4. *P*’s claim is credible to *Q*;
5. *Q* does not do *A*;
6. Part of *Q*’s reason for not doing *A* is to lessen the likelihood that *P* will bring about the consequence announced in (3).

This definition provides an initial (and as argued shortly, imperfect) set of necessary and sufficient conditions to determine whether, and if so when, an insurance company’s premium change is a coercive threat.

Following Nozick, we distinguish between *threats*, on the one hand, and *conditional offers* (hereinafter: offers) and *conditional (non-threatening) warnings* (hereinafter: warnings). To distinguish the three, one can provide the example of a factory owner facing the results of a referendum on whether employees should be represented by a union. Suppose that the owner warns the workers that, if the union wins the election, the factory will be closed (Nozick 1969, p. 453). This communication is a *warning* if the owner has reasons to close the factory independent of the intention to make the trade union alternative worse for the workers. For example, the factory would operate at a loss after satisfying all constraints unions are known to impose on such firms. The owner has an independent reason to close the factory; namely, he or she does not want to own an unprofitable business. A similar situation represents a *threat* if the owner *prefers non-unionized workers* and threatens to close the factory for strategic reasons: the owner has no conclusive reason to close the factory *but for* making joining the trade union less attractive for the workers.^7^ In a threat, the owner closes conditionally *in order to* worsen the alternatives for the workers. In a conditional warning, this is not true. The difference between threats and warnings ultimately comes down to this: in threats, the consequence produced by P is *intended* to make one of Q’s options less desirable, while warnings *describe* the consequence that makes such option less desirable, but the consequence is not intended to exist for that *purpose*. Clearly, when P threatens Q, consequences wanted by P are *intended* to make one of Q’s options less desirable as a way to influence Q’s actions.^8^

A review of conditions 1–6 reveals that they are ambiguous between threats, conditional offers or conditional warnings. This ambiguity is corrected by rewriting 3 as 3*:

3* *P*’s claim indicates that if *Q* performs *A*, then *P* will bring about some consequence that would make *Q*’s *A*-ing less desirable to *Q* than *Q*’s not *A*-ing, *Q’s available options are made worse (from Q’s point of view) by P’s decision to bring about such consequence, and the consequence is intended by P to make A-ing less desirable*.^9^

The first part of the addition (Q’s available options are made worse) and the last part (the consequence is intended by P to make A-ing less desirable) are intended to distinguish threats from offers and warnings, respectively. Concerning the former, to determine whether Q’s options are made better or worse as a result of P’s decision to bring about such consequence, we rely here on the *status quo* baseline, not the moral baseline, as argued above. One considers what is normal in the sense that it would be reasonably considered probable. We shall consider in the next section how this condition applies to the insurance case.

We now show that in the case of a threat of P against Q, as defined by 1–6 with 3*, Q’s *real freedom* (Van Parijs 1995) is reduced. A loss of real freedom follows from eliminating options *that the threat target values* from the set of all feasible options (real freedom, unlike *negative* freedom, also considers how an individual’s valuable options depend on the available resources, besides laws and other forms of coercion). Based on this definition, a subject who keeps access to all the old options is never made less free by the addition of further options. Moreover, a subject who faces a change of any existing options with a better option (from the subject’s own point of view) is not made less free. By contrast, coercive threats “take away the most preferred conjunctive choice from the option set” (Pugh 2020, p. 107). Threats reduce freedom by eliminating at least a preferred conjunctive choice, such as “working at this company and joining a trade union,” from the available options set, and substituting with a less valued one (such as working in this company and not joining a trade union or joining a trade union and not working in this company).

In the case of a conditional warning, the target of communication is simply made aware of this unfreedom, that is, the fact that the most preferred conjunctive choice from the option set is not possible. Crucially, however, this unfreedom does not exist because P *intends* to make one of Q’s alternatives (for example, joining the trade union) less desirable. For example, P’s announces that the factory will be closed if the workers are unionized, but P does not *intend* to close the *factory in order to* eliminate some workers’ most preferred conjunctive choice.

This combination of the impossibility of Q’s preferred conjunctive choice and the fact that P intends the consequence to make one of Q’s options less desirable is necessary for an announcement to be a threat. That is not true of warnings, where the consequence brought about by P does not have that purpose. It is also not true of *offers,* as we shall see next. As the argument at the end of this section shows, this definition makes coercion by threat a *pro tanto* wrong because it violates autonomy.^10^ As will be explained shortly, violating autonomy requires an *intentional* limitation of freedom, where freedom considers the options preferred by the subject of the threat.

One fundamental implication of this definition is that a consequence that P *must* produce, because it is legally or morally *required*, cannot be a threat. The reason is that the legal or moral requirement gives P *sufficient reason* to produce that consequence, so the consequence cannot be described as produced *for the sake of* making one option less desirable. That is also true when P, in fact, also wants to make one option less desirable. An example by Nozick (1969) helps clarify this point. Suppose that a teacher, who is *legally required* to assign grades based on a given scoring algorithm, tells the students, “if you do not study more, you will get bad grades.” This, intuitively, counts as a (non-threatening) warning if the teacher does not care about what his or her students do. But it still counts as a warning, not a threat, when the teacher desires that his or her students will study more. Furthermore, it still counts as a warning, not a threat, if a teacher has legal discretionary power to give the grades he or she believes appropriate but believes that he or she is *morally required* to give low grades to students who do not know the subject enough.

An *offer* differs from a *threat* because it announces a consequence that makes the target better off (Nozick 1969; Frankfurt 1988; Wertheimer 2014) (‘better off’ meaning here: offers an option that the target prefers). In the employer’s case, this would be an owner who offers to raise worker salaries, conditionally on workers not voting to join the union. In this case, the owner does not eliminate the option (being a union member, keeping one’s job), but adds a new conjunctive option (not being a unionized shop, getting an unexpected salary raise), including at least one welcomed element to the original option set. The option set of the target is intentionally manipulated, but the subject does not have less real freedom relative to the normally expected course of events.^11^

There is a debate in the literature on whether offers can be coercive, and, if so, what makes them so (Anderson 2017). It is not intuitive that conditional offers are coercive *as such*. If all conditional offers are coercive, then every employer coerces his or her employees (“I will pay you only if you work for me”), and every employee coerces his or her employers (“I will work for you only if you pay me”). The plausibility of this idea is related to our intuitions about human autonomy. Our assessment (which we cannot defend fully in this context) is that offers cannot violate the autonomy of the target, as long as it is still in the target’s power to ignore the enticement originating from the offer (Frankfurt 1988).^12^ Provided that the target has the real option to ignore the enticement originating from the offer, accepting an offer is not tantamount to being coerced.

Coercion by threats (as defined here) is *pro tanto* morally wrong because it violates a person’s *autonomy* (Blake 2001). The idea that autonomy deserves respect reflects one morally important aspect of how we treat (some) moral subjects,^13^ that is, as agents in their own right, showing proper respect for their full nature as agents. Autonomy “[…] demands that the set of options provide adequate materials within which to construct a plan of life that can be understood as chosen rather than as forced upon us from without” (Blake 2001, p. 269). Coercion violates the autonomy of its target because it intentionally reduces the target’s freedom (Blake 2001, p. 272) *for the sake of* influencing the chosen outcome. That is not true of either offers and warnings. Coercion is the result of a threat that has worked as intended.

## Distinguishing Threats from Warnings and Offers in the Insurance Domain

Suppose that many low-risk clients have accepted offers from insurtech start-up companies to share their data in exchange for better prices, also accepting terms and conditions where the clients get discounts if they act in ways that reduce risk, for example, exercise often, travel by bicycle, or visit their physicians regularly. The pool of the traditional insurer (which has been abandoned by the low-risk clients seeking discounts) now features a higher average risk. Some amount of adverse selection has taken place. The insurer is now operating below costs, as the average cost of claims went up. The insurer can either (a) raise the premiums to all clients to avoid insolvency or (b) offer cheaper surveillance products to attract low-risk clients and raise the premiums of other clients to compensate for the missing revenues. However, (a) will create an incentive for even more low-risk clients to search for alternatives, so the only sustainable solution is (b).

Announcing the intention to change the insured’s terms and conditions in the way specified by (b) *cannot* be considered a threat. The announcement should be framed as a *warning*: a mere description of the subject’s options, which the insurer gives to the client, *independently of the insurer’s intention to make it less desirable for the client to keep surveillance data private*. If the insurer truly *must* only permit less attractive options now, for example, raise insurance prices in the absence of certain data (for example, to avoid adverse selection), the insurer merely announces what the options are. Its announcement is a *warning,* not a threat.

The same announcement is a *threat* when the insurer raises prices *in order* to make the option of not sharing data less desirable for the client (for example, it is not necessary to have these data to prevent adverse selection and ensure financial sustainability). In the threat scenario, the insurer could have sustainably maintained traditional products (involving no digital surveillance) at the same (or normally expected) price while offering digital surveillance products to others at a lower price. But the insurer wants to build data assets and chooses to raise the premiums of all clients who do not share data from digital surveillance sources as a means to that goal.

A threat that can be avoided and does not coerce still counts as a threat. Suppose that a customer could avoid this threat by turning to a competitor with a preferable offer. There is no coercion in this case. Suppose that, instead, the customer is too lazy or busy to find an alternative. He or she decides to give in, wear a step-counter, and share its data. The choice still counts as coerced, despite the existing alternative options with a *different* insurer.

The difference between the insurer issuing a warning and the insurer who threatens to coerce corresponds to the difference between the two owners in the unionization example. It is the distinction between *threats* and *warnings.* What makes the options provided by a second insurer a threat is the fact that the insurer *intends* that the client cannot get traditional insurance at the normally expected price without sharing new data.^14^

Consider an insurer who leaves the premiums of existing clients intact but begins to offer insurance at a different, discounted price to those clients who agree to use a step-counter or a geo-locating mechanism and share their digital surveillance data. This pricing structure counts as an *offer,* not a threat, because the client gains further options from the insurer, and existing options are left intact. The client’s freedom expands, or at worst, does not shrink. Clients who switch from the old product to this new product cannot be said to have been coerced.

The slight complication introduced here is the idea of a *status quo* baseline not defined by constant prices. This baseline is not the relevant one, for example, because of inflation or changes in variable risk factors of the client that the existing insurance contracts already consider, such as age. The *status quo* baseline should be defined by reasonable expectations forming the background of any prior commercial agreement between client and insurer, in particular, those clearly stated in the contract. The *status quo* relevant to assess if the condition offered by a new product is a threat or an offer is not simply the premium actually paid in the past with old products. It is defined by the *reasonably expected status quo*: the premium that a *reasonable* client expects to pay, given the normally expected course of events. It is normal that premiums vary, reflecting both exogenous causes (inflation or variations in the overall expected cost of the risk pool) or endogenous ones (age and other risk factors).

In summary, necessary and sufficient conditions to call the manipulation of commercial products available to clients “coercion to accept digital surveillance insurance plans” are:

- The insurer wants the client to accept digital surveillance;
- the insurer has historically offered *insurance products* that do not involve *surveillance* (traditional products), with fairly predictable prices, grounding reasonable expectations of what the *normal* price for them would be;
- the insurer announces that it will raise the premiums of all those clients who have not purchased surveillance plans;
- the increased price of traditional products is higher than expected, even discounting for exogenous economic factors and fluctuating personal features known to affect premiums;
- it is not the case that, when market conditions have changed the risk pool of the insurer (for example, lower-risk individuals leaving the pool), the insurer has to raise prices for sustainability reasons;
- the insurer does not have to raise the price due to legal requirements or stringent moral obligations^15^;
- the insured purchases the product involving surveillance to avoid paying a higher premium.

In the example above, coercion violates the client’s autonomy to accept or avoid digital surveillance by the insurer.

The distinction underpinning the threat/warning distinction depends on the distinction between a discretionary (but still profit-affecting) business decision (intended to make refusals to share data less desirable for the client) and a decision that a business *must* make. One may doubt that the underlying distinction is tenable. In many cases, it will be hard to determine if a choice is avoidable.

Yet we believe the distinction is tenable, even though it is often difficult to apply to concrete cases. A parallel will be made with assessments of an agent’s virtue for which no clear-cut criteria exist and require examining habitual dispositions of the agent, involving behavior about the agent’s past, that are often hardly accessible to casual acquaintances (Everett et al. 2006). Similarly, the complexity of a company’s life, and the path-dependency of its choices and strategies, will often make it difficult to assess if a price increase of non-surveillance-based options is unavoidable (for example, for prices to be actuarially fair and avoid adverse selection in the insurance pool) or intended to produce an effect on the client’s willingness to contribute to insurer data assets. In some cases, only insiders will be able to tell. In many cases, even insiders will disagree on whether a choice to raise the prices of some products was the only, or best, option enabling long-term stability and the repayment of all claims and obligations in a competitive, transforming environment.

As with actions realizing virtues, we can point to the existence of some clear-cut cases, those in which the executives of a company agree that a certain choice is either optional or required.^16^

Another objection is that some businesses may view profit maximization as an imperative and the only relevant moral imperative for firms (Friedman 2007). If this view is correct, profit-maximizing insurers cannot threaten their clients. Notice, however, that the view “*if* it is imperative for firms to maximize their profits, profit-maximizing insurers cannot threaten their clients” is not incompatible with the view defended here because it is simply an application of this view. Our view is logically compatible with the view that profit maximization is an imperative because, if that contentious general premise is true, then the proposal to raise the prices for clients who do not share data is a conditional warning *in the case* that is necessary to maximize profits. Even in that case, threats are possible. For example, suppose that raising the premium of clients who do not share their data is *not* a profit-maximizing strategy. Management knows this, and yet it raises premiums to build data assets. Managers do this for the sake of signaling that the company is aggressively pursuing innovative strategies, which is expected to cause a short-term rise in stock prices, resulting in an end-of-year bonus. Managers do not increase premiums for the sake of maximizing long-term shareholder value but their own short-term gain. The conditional proposal should be classified as a threat, even if the contentious assumption above about profit maximization is granted. On the other hand, if there is no imperative for firms to maximize profits (Freeman 2010), there could be different circumstances in which companies threaten clients.

## Privacy, Autonomy, and the Harm of Being Coerced into Surveillance

In this paper, we define the privacy of a person X as a restriction for third persons to access the self of X (including information about the self). We focus on privacy when it is valued as a constituent or contributor to the spontaneity, authenticity and autonomy of X. Our definition builds on traditional accounts of privacy (Bok 1984; Bloustein 2003; Gavison 1984) that are widely discussed in the privacy literature. We note that our definition is incompatible with others that are considered equally important (e.g., Solove 2002; Moore 2003; Nissenbaum 2004). Moreover, our definition covers less ground (and not necessarily the same ground) as social-norms-based (contextual) conceptions of privacy (Solove 2002; Moore 2003; Nissenbaum 2004). We ignore the latter because we do not need to claim that our conception of privacy is the only right one. We do not appeal to privacy as an independent normative premise of this argument independent of autonomy, spontaneity and authenticity. We simply use ‘privacy’ as a shorthand for a condition of “limited access to the self (including information about the self)” when we discuss how this condition is affected by a coerced choice and affects other goods. Notice that autonomy plays two distinct roles in our argument: first, autonomy is that which is violated when the client is coerced to give up privacy. In this sense, the distinct wrong of coercion is that it fails to respect autonomy, and this wrong is independent of the value of privacy. (Coercion would also be wrong as the violation of the autonomous choice to play tennis.) Second, autonomy gives the individual *reasons* to value his or her own privacy (a condition of restricted access to the self) instrumentally. In this case, privacy is valued as a means to autonomy. Arguably, the goods of spontaneity and authenticity provide similar reasons. We develop both arguments below.

Some individuals value privacy *for its own sake.* When this happens, privacy can be an aspect of an autonomously (or spontaneously, or authentically) chosen life. In this respect, privacy is conceived *in itself* the *object* of a choice, which can be autonomous, spontaneous, and authentic, not a *means to* or *enabling condition of* further choices. For example, the intrinsic value of privacy may amount to this: individuals may not want to expose certain aspects of their lives to other people, irrespective of the consequences. Individuals may be uncomfortable with the idea of being seen, heard, perceived, or recorded, even by a machine, in certain circumstances (Westin 1967).

Furthermore, privacy is valuable *instrumentally*, for the sake of making autonomous (or spontaneous, or authentic) choices about other aspects of life. In this case, privacy is the *condition of* or *means to* a choice about something other than privacy. The instrumental value of privacy is explained more easily by presenting some common consequences of lacking it. People lacking privacy are more vulnerable to costs imposed by others if they behave in particular ways. There are also indirect costs for spontaneity and authenticity if individuals act less authentically or spontaneously because they fear such costs. If authenticity or spontaneity are enabling conditions of human dignity and individuality (Bloustein 2003) or enabling conditions of authentic friendship and love, and if friendship and love are objective elements of well-being (Griffin 1986), authenticity and spontaneity are also valuable for those reasons. In other words, even when privacy is not valued for its own sake, it may be valued as a condition of something else, which is.

So, the first argument against coercion to adopt digital surveillance for insurance purposes is that it disrespects a preference for privacy. When that desire expresses the autonomy of the individual, this is *pro tanto* morally wrong, as argued in Sect. 4. The second argument against coercion to adopt digital surveillance is that it destroys a condition that protects autonomy and favors spontaneity and authenticity. This argument is *independent* of digital surveillance being coerced. The result of losing privacy is morally bad (*pro tanto*) in that it (1) generates further risks of coercion, which is always *pro tanto* wrong, and (2) reduces the opportunity for express autonomy, authenticity and spontaneity in other choices. The rest of this section deals with arguments (1) and (2).

Let us start by considering: (1) the loss of privacy as exposure to potential coercion. Individuals are exposed to coercion because of a lack of privacy resulting from their acceptance of digital surveillance. A person who is willing to constantly generate and record some digital phenotype, such as wearing a fitness tracker with GPS, is exposed to new threats. For example, once an insurer has surveillance data, the insurer may threaten to raise premiums (relative to the normally expected prices) if the client does not adopt some required risk-reduction activities or is not willing to demonstrate them through accessible digital phenotypes.

Further violations of autonomy result from threats that *other* agents can make when they know that digital phenotypes are generated and recorded. For example, an employer may threaten the employee, or a husband may threaten the wife, to be given access to data shared with insurers. For example, the employer may be able to mine these data to infer how much time an employee spends in front of a computer, and a jealous husband may be able to know how much time his wife spends outside the home. Once that threat is accepted, further threats exploiting the surveilled activities are enabled; for example, the employer and the jealous husband may coerce the employee and the wife to act in desired ways through further threats. By showing that a privacy loss generated risks of further coercion when it was uncoerced, we show that non-coercive processes leading to a loss of options for privacy-valuing clients generate risks of further autonomy violations that are potential *pro tanto* wrongs. This is the premise of the argument provided in “Privacy erosion from non-coercive insurance market transactions” section, dealing with privacy erosion from non-coercive market transactions. In addition to being instrumentally valuable as a protection of autonomy, privacy is valuable as a means to spontaneity and authenticity. Individuals who accept digital surveillance because of threats, offers, or warnings may later find out that they are no longer able to act spontaneously or authentically. Such long-term harmful effects of losing privacy may be hard to foresee when clients autonomously choose surveillance.

One could object that some clients are made better off by such “offers”, that is, cases in which sharing data lowers the premium, while the refusal to share data raises it, relative to the expected status quo. These, as a result, do not count as *threats* against them. Some clients have nothing to hide because they know they are low risk (they are already very physically active, for example). They may not have an intrinsic aversion to being tracked, as long as observation by actual human eyes is ruled out. And, for this client, the appeal of a discount may be sufficient to motivate him or her to share digital surveillance data with the insurer.

In response, let us consider whether even clients who do not value their privacy intrinsically may have reasons to value their privacy instrumentally as a protection from the *counterfactual interference* of the insurer (De Bruin 2010), which amounts to protection of their freedom (De Bruin 2010).^17^

To explain this value of privacy, consider a client who values the option of *paying a low premium* and does not value privacy intrinsically. That is, this client does not dislike being surveilled, as such. A proposal framed as “you pay less than before if sharing digital surveillance data and you pay more than before if not sharing it” is an offer because the proposal augments the client’s *valued* options, that is, provides lower premiums and does not prevent access to any valued option, as the client does not care whether surveillance is in place. So, this arrangement does not count as a threat.

Let us consider how this changes the client’s access to other options in counterfactual scenarios. For example, before being digitally surveilled, the client could freely decide to stop doing many physical activities without suffering a rise in premiums. Now, after accepting digital surveillance, the client who decides to no longer do physical activities must pay a higher price reflecting his or her increased risk. The previously existing option of not doing frequent physical activities is now counterfactually *blocked by a threat*. It is not blocked in the sense that the previously existing non-digital-surveillance option is no longer offered, and it is not blocked in the sense that it is made inaccessible by the direct imposition of physical force (that is, no one physically takes the client to the gym). Yet, the option is now counterfactually blocked by *coercion*: were the client to stop doing a lot of physical activity with a surveillance-based option, he or she would be required to pay a higher premium, were the client to return to a non-surveillance-based option, he or she would be required to pay a higher premium. When this rise in prices is intended by the insurer to make those options less desirable for the client (as required by 3*), the *counterfactual interference* of the insurer takes the form of a counterfactual threat.^18^

Summing up: a deal where sharing data lowers the premium, but refusing to share data raises it, relative to the expected status quo, counts as an offer for a client who does not value privacy intrinsically, exercises frequently, and is sufficiently motivated to share data by the discount. And yet, it can reduce the client’s *freedom* (De Bruin 2010). This freedom loss occurs when the digitally surveilled client would (counterfactually) be threatened to avoid physical inactivity. More generally, the example shows that the instrumental value of privacy *also* consists in protecting the client’s freedom understood as freedom from counterfactual interference (De Bruin 2010).

According to the first line of argument, the threat of the insurer is wrong because, when accepted, it amounts to coercion, which is *pro tanto* wrong, being a violation of the client’s autonomy. According to the second line of argument, the increased cost of privacy-friendly options (whether coerced or uncoerced) is *pro tanto* harmful when it enables the insurer or third parties to coerce the client. In the latter case, the increased cost of privacy makes protecting autonomy, spontaneity and authenticity costlier and it reduces the client’s freedom. The next section examines a plausible scenario of the evolution of insurance models favoring the adoption of digital surveillance through non-coercive means.

## Privacy Erosion from Non-coercive Insurance Market Transactions

In this section, we describe a possible, and at least not unlikely, social process that reduces freedom, that is, eliminates valued conjunctive options for privacy-valuing individuals. The case we describe here deserves to be discussed in detail because it does not qualify as coercion and yet causes a freedom loss. This loss of freedom makes privacy costlier to obtain or maintain, and in this way, generates further threats against autonomy. This example helps us to answer the question of whether, in the absence of coercive threats by insurers, the introduction of digital surveillance is morally problematic, and at least *pro tanto* morally wrong*,* and if so, why.

We shall suppose here that the first insurer to introduce personalized premiums based on digital surveillance is a new market player offering *only* digital surveillance plans. Because such a player has no pre-existing clients on traditional plans, this insurer cannot be accused of threatening clients into sharing data. The insurer may tempt clients to share their data with strong discounts, but according to our analysis, that only counts as an *offer*, not as a *threat*. From the client’s perspective, a new company offering new products can only represent a gain in freedom: it is either a benefit or at worst neutral. So, no coercion via threats is involved at this stage.

Now suppose that new players entering the market obtain the former lowest-risk customers from traditional insurers. Thus, traditional insurers will either have to raise the premiums to all clients or also start discriminating (in, let us suppose, *actuarially fair* ways) between high-risk and low-risk clients, in ways that require collecting surveillance data to discern more granular risk pools. This requirement means that, eventually, the clients may end up receiving the following offer: either pay higher premiums than before or share more data than before. This offer resembles a threat, and certainly, it is not an offer because it involves worse options than the status quo. At closer inspection, this is not a threat either, if the company, as described, is merely adjusting its prices to avoid adverse selection and the risk of insolvency.^19^ In the scenario we are considering, traditional insurers at a certain stage *must adopt* this policy to survive and fulfill their ethical duties to their clients*.* The offer, therefore, qualifies as a *warning* under the classification we propose.

The process we have described at no point involves threats by insurers to obtain more data. However, the market reduces the *freedom* of those clients who are, as a result, exposed to further (counterfactual) threats*.* This process starts with new players *offering* lower prices, clients accepting new forms of surveillance, and ends up with traditional insurers *warning* clients who do not accept such surveillance that premiums will become higher than they were reasonably expected to become.

The end result of this process is that privacy-valuing customers suffer a loss of freedom that is achieved without coercion by insurers. When assessing this scenario, one can focus on the *process* or the *outcome*. The *process* is not morally objectionable from the viewpoint of autonomy because the autonomy of the client is not violated by conditional offers and conditional warnings. The *outcome* of the process is, however, morally objectionable, at least *pro tanto,* because it exposes the individual to further threats, so it makes it harder for individuals to resist further attempts of coercion by insurers or other agents. Digital surveillance creates risks of *pro tanto* moral wrongs. In a sense, the scenario described here is inimical to autonomy. But the client’s autonomy is not violated by the insurer’s offer (warnings) for clients accepting (refusing) surveillance. Rather, the effect of the insurers’ and clients’ spontaneous, uncoordinated, and often autonomous actions make autonomy costlier to protect for insurance clients in general. Moreover, the outcome of the process is also morally objectionable because it increases the cost of protecting authenticity and spontaneity, if these are valuable, as argued in Sect. 5. This provides insurers, clients, and regulators *pro tanto* reasons to avoid this outcome that are independent of the moral wrongness of coercion by insurers to accept digital surveillance.

## Further Research

In what follows, we highlight possibilities for further ethical research that result from this analysis of privacy and autonomy outcomes resulting from threats, offers, and warnings leading clients to accept digital surveillance by insurers.

One question to research is whether, given a collective responsibility of insurers to generate an equilibrium in which protecting autonomy, authenticity, and spontaneity becomes costlier, insurers have moral duties to avoid contributing to it. Another question is whether consumers have a moral duty to avoid taking conditional offers for cheaper insurance in exchange for data. For both questions, if the end result of the process is morally wrong all-things-considered, one could explore a rule-consequentialist argument that prohibits certain choices that would contribute to such outcomes.

A third question is whether regulators have duties to prevent this process from happening, that is, all-things-considered reasons to interfere with market transactions, through sanctions. Notice that sanctions also imply a loss of freedom. For example, enforcing regulations against conditional *offers* reduces the freedom of clients who favor or are indifferent to digital monitoring and would otherwise have made small savings. A utilitarian regulator should prohibit conditional offers only if the loss of freedom for some, accompanied by the gain of freedom by others, produces a utility gain overall. We do not have any empirical evidence backing one or the other alternative.

From the viewpoint of autonomy-based liberalism (Blake 2001), it may be argued the expansion of digital surveillance undermines general conditions for individual autonomy that ought to be preserved for all individuals, even those who do not value it. Such a regulator should claim that even individuals who voluntarily adopt digital surveillance face a serious risk of losing their autonomy, spontaneity, or authenticity with respect to *other* choices made under insurance surveillance. The latter is the greatest wrong. Protecting the general conditions for autonomy from a potentially indefinite expansion of digital surveillance may justify the interference of the state with (even non-coercive) market exchanges (Blake 2001; Raz 1986; Rawls 1980)*.* A liberal regulator, in this perspective, has reasons to limit the freedom of clients who want digital surveillance, that is, to prohibit conditional offers involving digital surveillance, when facing a serious risk that privacy-friendly products will become more expensive for those who want them at a later stage.^20^ Our analysis reveals the following dilemma: is the certain immediate freedom loss of consumers who do not value privacy, implied by preventing the trade between privacy and small savings on premiums, a morally worse state of affairs, from the point of view of autonomy, than the risk of uninterrupted incremental exposure to digital surveillance (as argued here, a risk factor for further coercion) that this regulation is supposed to prevent? We hope scholars from various disciplines will contribute to answering this question.

## Conclusion

In this essay, we have argued for three theses involving insurance threats, privacy, and autonomy. First, coercion to share private data with insurers is *pro tanto* wrong because it is a violation of the client’s autonomy. Second, we maintain that being coerced into sharing data for insurance surveillance exposes the client to further threats and autonomy losses, and consequently, to the risk of living a less autonomous, spontaneous, and authentic life. Third, we point out that regulators may have reasons to oppose the contraction of freedom generated by digital surveillance, irrespective of whether clients were coerced into accepting it. All our arguments are *pro tanto*. One could accept our *pro tanto* argument and still argue that an unregulated market is *ultima facie* morally justified, for example, on efficiency and fairness grounds. Our analysis of the outcomes of non-coercive market transactions leading to the acceptance of digital surveillance opens further moral questions that there is not space here to address. We hope that this conclusion will stimulate other scholars to further research the topic.

## Footnotes

1 For example, the NGO Privacy International calls for challenging corporate data exploitation. If that is meant as preventing industries from “look[ing] for patterns and similarities, […] and mak[ing] decisions about us” (Privacy International n.d.), this could, in principle, apply to any form of risk assessment performed by insurers.

2 See, for instance, the question of ‘conditionality’ in the satisfaction of the informed consent criterion in the General Data Protection Regulation that consent shall be “freely given” (Art. 29 Working Party 2018).

3 The distinction between even *violent* coercion by threats and coercion by direct force is established, philosophically. For example, Aquinas discussed two ways in which violence undercuts voluntariness: (a) when used directly against the body, “a man may be dragged by force: but it is contrary to the very notion of violence, that he be dragged of his own will” (Aquinas 1920, sec. I.II Q6 A4), and (b) when a threat of violence causes one to act from fear, or to avoid that violence, which, in Aquinas’ view, does not make the act fully involuntary (Aquinas 1920, sec. I.II Q6 A6; see Anderson 2017, sec. 1.2).

4 Nozick (1969) claims that, when the *status quo* and the *moral* baseline diverge, we should adopt the baseline that the coerced individual himself or herself would prefer to be adopted. Our argument for the *status quo* baseline can be regarded as an argument that the client would prefer a *status quo* baseline.

5 It may still be claimed that Wertheimerian threats arise out of a generic entitlement of individuals to be insured, without a corresponding perfect duty by insurers to be the agents providing insurance. If that generic entitlement to be insured is understood as an entitlement to social insurance, we object that socializing all insurance products currently in the market (that is, beyond health and unemployment or a few other forms of insurance meeting people’s urgent needs) does not appear to be plausible. In other words, it is implausible that the state should tax citizens to socialize the insurance costs of the theft of expensive phones, or holidays in dangerous places.

6 These conditions are not literal citations of those Nozick discusses but are intended to capture the core elements of his account.

7 Or at least to produce a profit equivalent to the wage earned to manage a similar company plus an appropriate risk premium for the equity invested in the company.

8 Even if this intention may not be evident in the way they are announced.

9 So, why not define a threat as harm induced “in order to get Q to do A”? Because, in the example of the researchers threatening the life of research subjects unless they hand over a wallet, for the sake of studying how they respond (Nozick 1969), the intention is not that Q is coerced to do A (for example, pay the money) but that Q responds in any way to the threat, so that Q produces interesting data. This still counts as a threat.

10 Kushner (2019) has defended the claim that coercion is a *pro tanto* wrong. Kushner’s view is *compatible* with our view of coercion; that coercion is a *pro tanto* wrong *because* it violates a person’s autonomy. His account does not require the autonomy-based account. Kushner only aims to be agnostic with respect to substantive explanations of the wrongness of coercion.

11 Suppose that subject P can go to two ice-cream shops: A = [strawberry, pistachio, chocolate] and B = [strawberry, vanilla, banana]. P values pistachio and chocolate but does not value vanilla or banana. We consider P’s freedom to be greater in A, even though the two ice-cream shops contain the same number of options, because the options in ice-cream shop A are those that the subject values.

12 This may be false if such thing as an ‘intrinsically irresistible’ offer exists, or if the subject is deprived of the minimal psychological conditions necessary to be in control of his or her choice when facing the offer. For example, P makes it impossible or really hard for Q to focus on anything but the enticement, by invading P’s environment with a communication that reminds P of the offer. This would not turn the offer into a threat, but it would violate autonomy nonetheless. We believe that this is an extreme case and not very relevant to the case at hand.

13 We do not rule out the possibility of moral subjects that are not human agents and that are moral subjects by virtue of their sentience and their capacity to feel pain and pleasure.

14 In practice, it will be difficult for clients to determine if a given announcement states, in reality, a warning or a threat. The insurer has a reputational reason to mask threats as mere warnings.

15 When legal or moral requirements provide the insurer with conclusive reasons to raise premiums, the announced raise in prices is a warning. This can be judged by analogy with the example of the teacher in the preceding section.

16 Moreover, certain forms of path-dependency can be assessed morally, and the definition of threats used here can be adapted as needed to include those more complex cases. For example, if at time *t* you can predict that by doing F at *t* you will put yourself in a position at *t* + *n* where you *must do* G (e.g., raising the premium of a customer who does not share data), G counts as *avoidable* to the extent that any feasible alternative to F not leading to G (compatible with the survival of the firm and overriding moral and legal duties) exists at *t*. It is not our purpose here, however, to provide a theory that solves all moral puzzles of path-dependency.

17 De Bruin (2010) characterizes this freedom as “negative”, defined as the concept according to which “I am unfree to perform some action, A, if someone interferes with my performance of A or if someone has the disposition to interfere with my performance of A if I were to attempt to perform A” (p. 511). The freedom in question here can be labeled “negative” only if *interference* in this definition includes *threats*. It cannot be labeled “negative” if negative freedom reflects only *physical impossibility* and excludes threats (Steiner 1994).

18 Unless it is something the insurer *must* do, in which case it counts as a *warning.*

19 Or maintain reasonable profitability margins to avoid de-capitalization, preventing long-term operations.

20 Or, as an alternative, has reasons to coerce insurers to offer traditional products at the price that would have been possible in the absence of surveillance.
